# Digitizing the Informed Consent Process: A Review of the Regulatory Landscape in the European Union

**DOI:** 10.3389/fmed.2022.906448

**Published:** 2022-05-25

**Authors:** Evelien De Sutter, Janos Meszaros, Pascal Borry, Isabelle Huys

**Affiliations:** ^1^Division of Clinical Pharmacology and Pharmacotherapy, Department of Pharmaceutical and Pharmacological Sciences, KU Leuven, Leuven, Belgium; ^2^Centre for Biomedical Ethics and Law, Department of Public Health and Primary Care, KU Leuven, Leuven, Belgium; ^3^Centre for IT and IP Law (CiTiP), KU Leuven, Leuven, Belgium

**Keywords:** electronic informed consent, ethics, privacy, General Data Protection Regulation, Clinical Trials Regulation, clinical trial

## Abstract

**Background:**

Rapid technological advancements are reshaping the conduct of clinical research. Electronic informed consent (eIC) is one of these novel advancements, allowing to interactively convey research-related information to participants and obtain their consent. The COVID-19 pandemic highlighted the importance of establishing a digital, long-distance relationship between research participants and researchers. However, the regulatory landscape in the European Union (EU) is diverse, posing a legal challenge to implement eIC in clinical research. Therefore, this study takes the necessary steps forward by providing an overview of the current regulatory framework in the EU, relevant to eIC.

**Methods:**

We reviewed and analyzed the key EU regulations, such as the EU General Data Protection Regulation (GDPR) and the Clinical Trials Regulation (CTR). We investigated the legality of eIC in several EU Member States, Switzerland, and the United Kingdom. To this end, we contacted the medicines agencies of various countries to clarify the national requirements related to the implementation and use of eIC in clinical research. Our research was complemented by comparing the legal acceptance of eIC between the EU and the United States.

**Results:**

In the EU, a distinction must be made between eIC for participation in clinical research and eIC for processing the participants’ personal data, complying respectively with requirements laid down by the CTR and the GDPR. On a national level, countries were classified into three groups: (1) countries accepting and regulating the use of eIC, (2) countries accepting the use of eIC without explicitly regulating it, and (3) countries not accepting the use of eIC. As a result, the regulation of eIC through laws and guidelines shows a large variety among EU Member States, while in the United States, it is harmonized through the Code of Federal Regulations.

**Conclusion:**

Various requirements must be considered when implementing eIC in clinical research. Nevertheless, requirements across the EU Member States may differ significantly, whereas, in the United States, efforts have already been made to achieve a harmonized approach.

## Introduction

The principle of obtaining informed consent (IC) from participants as a prerequisite to participate in clinical research has initially been embedded in the Nuremberg Code and Helsinki Declaration (1–3). The practice of IC is also enshrined in guidelines for Good Clinical Practice (GCP). The GCP guideline of the International Council for Harmonization of Technical Requirements for Pharmaceuticals for Human Use describes ethical and scientific requirements when designing, conducting, recording, and reporting clinical trials involving human subjects. According to this guideline, IC is “*a process by which a subject voluntarily confirms his or her willingness to participate in a particular trial, after having been informed of all aspects of the trial that are relevant to the subject’s decision to participate*” (4). IC may also serve as a legal ground for processing research participants’ personal data, according to the basic principles embedded in the European Union (EU) General Data Protection Regulation (GDPR) (5, 6). In the GDPR, IC is one of the legal bases for processing personal data^1^.

The IC process has historically always been documented on paper forms. However, digital technologies are reshaping this process, since electronic informed consent (eIC) could offer various advantages compared to paper-based IC forms (7). For instance, eIC could include multimedia components such as audio and video to present information in an interactive and engaging way (8). Moreover, eIC could include a personalized communication interface and facilitate ongoing communication with research participants. Nevertheless, face-to-face contact between the participants and the research team remains crucial to establishing a relationship of trust, and it is necessary for individuals who do not have access to technology or lack digital literacy (8, 9). Various initiatives have already implemented innovative types of consent and reported on the use of dynamic consent, a type of eIC, in biobanking and epidemiological research (10, 11). Dynamic consent is primarily developed to provide participants with more control over the future use of their data and samples (11). It enables participants to interact with the interface to manage their decisions on the use of their personal data or samples in research studies over time and thus, to have more control over their involvement in research (10, 12). Additionally, dynamic consent may increase transparency by providing an overview of participants’ data usage or by feeding back study results (13).

In 2021, the European Medicines Agency (EMA) published a draft guideline on computerized systems and electronic data in clinical trials. As described in this guideline, eIC refers to “*the use of any digital media (e.g., text, graphics, audio, video, podcasts, or websites) to firstly convey information related to the clinical trial to the trial participant and secondly document informed consent via an electronic device (e.g., mobile phones, tablets, or computers)”.* As the EMA highlighted, investigators need to pay attention when using electronic methods since it might discriminate against people who are not comfortable using this kind of technology, potentially leading to bias in clinical research. Therefore, alternative methods for providing information and documenting IC should be available for those unable or unwilling to use electronic procedures. The EMA emphasized that any sole use of eIC should be justified and described in the protocol (14).

Despite the increasing interest in eIC, its adoption has been hampered due to various reasons. The most critical issue is the legal acceptance of eIC in clinical research (8, 9, 15). Concerns have been raised regarding whether eIC would comply with local regulations (8, 15). Moreover, it was reported that legal requirements, related to eIC, may differ across countries. For example, some countries require participants’ wet-ink signature and do not accept the use of electronic signatures to provide IC for study participation (9). To this end, this manuscript aims to analyze the current regulatory framework relevant to (electronic) IC, examining how this framework could be further developed to facilitate eIC implementation in clinical research.

## Methods

We reviewed and analyzed the key EU regulations, such as the GDPR, Clinical Trials Regulation (CTR), and the Regulation on electronic identification and trust services for electronic transactions in the internal market (eIDAS Regulation). Moreover, we complemented our analysis showcasing national requirements related to the implementation and use of eIC in clinical research. For this purpose, we contacted the medicines agencies of several countries and described the various approaches currently used. This research was complemented by comparing the legal acceptance of eIC in the United States (US). Furthermore, recommendations were suggested to promote the implementation of eIC in clinical research.

## Results

### Electronic Informed Consent in European Regulations

#### Informed Consent for Participation in Clinical Research

The CTR, an EU-level binding legislative act, aims to harmonize the conduct of clinical trials throughout the EU and increase transparency in this field (16, 17). IC, which has a central relevance within the CTR, is defined as “*a subject’s free and voluntary expression of his or her willingness to participate in a particular clinical trial, after having been informed of all aspects of the clinical trial that are relevant to the subject’s decision to participate or, in case of minors and of incapacitated subjects, an authorization or agreement from their legally designated representative to include them in the clinical trial*”. Pursuant to Article 29 of the CTR, requirements are defined to obtain valid IC. IC must be written, dated and signed by the research participant and a member of the investigating team performing the interview, in which the necessary study-related information is conveyed to the participant (17). As a result, the CTR does not offer specific guidance for the use of eIC for participation in clinical trials.

#### Informed Consent as a Legal Ground for Processing Personal Data

The GDPR defines personal data as any information that can be directly or indirectly linked to an individual^2^. Special categories of personal data refer to sensitive information, such as health data, and it is restricted to process this type of data. According to the GDPR, data processing must be based on one of the legal grounds described in Article 6 and one of the conditions in Article 9 in the case of sensitive data. IC is one of these legal bases for the lawful processing of personal data. The consent of the data subject is specified as “*any freely given, specific, informed and unambiguous indication of the data subject’s wishes by which he or she, by a statement or by a clear affirmative action, signifies agreement to the processing of personal data relating to him or her*” (6). The GDPR clarifies that consent may be given by an oral or a written statement, including by electronic means^3^. The human subjects’ IC related to data processing can be obtained together with their IC for research participation. However, the request for IC related to data processing should be clearly distinguished from IC for research participation (6, 18).

The GDPR aims to foster transparency regarding the way human subjects’ data are processed (6, 19). Therefore, clear and plain language must be used when informing human subjects about the purpose and legal basis for data processing, the categories of recipients, the contact details of the controller and the data protection officer and if applicable, the transfer of personal data to a third country or international organization. Additionally, information must be conveyed about other aspects, such as the period of data storage and the data subjects’ rights (e.g., the right to be forgotten) (6).

#### The Interaction Between the General Data Protection Regulation and the Clinical Trials Regulation

The CTR provides that EU Member States shall apply the GDPR to the processing of personal data carried out in the framework of the CTR^4^. Therefore, protecting the participants’ privacy is one of the conditions to conduct a clinical trial^5^. The GDPR also refers to the relevant legislation applicable to clinical trials^6^. Therefore, both regulations apply simultaneously, and the CTR constitutes a sectoral law containing specific provisions relevant to data protection. Regarding the legal basis for the processing of personal data during the lifecycle of a clinical trial, the European Data Protection Board (EDPB) considers distinguishing between two main categories of processing activities (20):

(1) *Processing operations related to reliability and safety purposes*: these processing activities do not necessarily have to rely on consent. Processing for reliability and safety can be covered by the legal grounds of “legal obligation”^7^ or “public interest” in the case of sensitive data^8^. As noted by the EDPB, several activities envisaged under the CTR satisfy the conditions for the applicability of this legal basis. For instance, archiving of the clinical master file^9^ or disclosure of clinical trial data to the competent authorities for an inspection^10^.
(2) *Processing operations purely related to research activities* in the context of a clinical trial cannot be based on a “legal obligation”. Depending on the trial and the concrete data processing, they may either fall under the data subject’s explicit consent,^11^ a task carried out in the public interest^12^ or the legitimate interest of the controller^13^.

#### Informed Consent in the Clinical Trials Regulation and General Data Protection Regulation: Not the Same

As the EU Commission’s Directorate-General for Health and Food Safety highlighted, the IC under the CTR must not be confused with the notion of consent as a legal ground for the processing of personal data under the GDPR (21).

Under the CTR, IC serves as an ethical standard and procedural obligation,^14^ addressing the ethical requirements of research involving humans derived from the Helsinki Declaration. The IC under the CTR functions as a measure to protect the right of human dignity and integrity of individuals under the Charter of Fundamental Rights of the EU (22). Therefore, IC under the CTR is not conceived as an instrument to comply with data protection requirements. As the Directorate-General pointed out, IC in the context of the CTR is a safeguard for the participants, and not a legal basis for data processing (21).

Under the GDPR, consent must be specific, informed, unambiguous, and freely given. Moreover, the consent must be explicit when special categories of data, such as health data, are processed^15^. From these requirements, the “*freely given*” may be the most challenging one in the GDPR, since it implies real choice and control for the data subjects (23). Therefore, IC cannot be a valid legal ground for data processing where there is a clear imbalance between the data controller and the data subject^16^. The EDPB clarified that imbalance of power might exist wherever there is “*a risk of deception, intimidation, coercion or significant negative consequences (e.g., substantial extra costs) if the data subject does not consent. Consent will not be free in cases where there is any element of compulsion, pressure, or inability to exercise free will*”. The primary examples for these situations are when the employer, public authority, or doctor ask for consent from the employee, citizen or patient to process personal data (23). In the case of clinical trials, the imbalance between the investigator and the participant might occur. Therefore, IC may not always be the proper legal ground for processing personal data in clinical trials (Table 1). Thus, for processing personal data, other legal bases may be necessary, such as “public interest” or “legitimate interest” (18). The CTR also addresses the issue of imbalance, highlighting “…*whether the potential subject belongs to an economically or socially disadvantaged group or is in a situation of institutional or hierarchical dependency that could inappropriately influence her or his decision to participate*”.^17^ Additionally, the EDPB considers that this will be the case when a participant is not in good health condition (18). However, ethics committees can allow consent in these imbalanced situations, with specific safeguards, such as supporting the patients’ decision-making by discussing their choice with a trusted adult or relative, especially when potentially vulnerable groups are involved (24).

**TABLE 1:** Processing health data in imbalanced/dependent cases.

Imbalanced/dependent situations (e.g., employer-employee, physician-patient, public authority-citizen)	
**Clinical Trials Regulation**	**General Data Protection Regulation**
Consent might be possible (e.g., with ethics committee review and specific safeguards)	Consent may not be the proper legal ground, since not freely given. Another legal ground is necessary for processing personal data (e.g., public interest)

The withdrawal of IC under the CTR and the GDPR should also not be confused. The CTR clarifies that participants may withdraw from a clinical trial at any time by revoking their consent. This withdrawal shall not affect the activities already carried out^18^ before the withdrawal^19^. Under the GDPR, there is also a possibility for data subjects to withdraw their consent at any time,^20^ and there is no exception from this rule in the case of scientific research (25). The GDPR requires that consent can be withdrawn by the data subject as easily as giving consent; thus, eIC is a convenient method to fulfill this requirement^21^. The GDPR does not imply that giving and withdrawing consent must always be done through the same action. As a result, it is possible to withdraw a paper-based IC electronically. However, when the participant’s IC was acquired electronically, he or she should be able to withdraw it as easily as giving it (23). In this case, similarly to the CTR, the withdrawal of consent shall not affect the lawfulness of processing the data based on consent before the withdrawal. Once the consent has been withdrawn, the data controller must stop data processing and ensure that the data are deleted or anonymized, unless the data can be processed on another legal ground (6, 17).

#### Signing Informed Consent Electronically

Next to providing study-related information to research participants electronically, eIC also refers to documenting their IC via an electronic device (14). To this end, various types of electronic signatures may be used. The eIDAS Regulation establishes a legal framework for electronic services, including electronic signatures, across the EU Member States. The main objective of this Regulation is to “*remove existing barriers to the cross-border use of electronic identification means used in the Member States to authenticate, for at least public services*”. Therefore, the Regulation does not create a general obligation to use electronic signatures. EU Member States “*remain free to use or to introduce means for the purposes of electronic identification for accessing online services*”, in particular in the private sector^22^. The Regulation only provides a legal framework for electronic services and encourages the use of them. According to Article 3 of the eIDAS regulation, an electronic signature is defined as “*data in electronic form which is attached to or logically associated with other data in electronic form and which is used by the signatory to sign*” (26). Another signature type defined in this Regulation is an advanced electronic signature. More specifically, an advanced electronic signature is an electronic signature that is “*(a) uniquely linked to the signatory, (b) capable of identifying the signatory, (c) created using electronic signature creation data that the signatory can, with a high level of confidence, use under his sole control, and (d) is linked to the data signed therewith in such a way that any subsequent change in the data is detectable*”^23^. In addition, the eIDAS Regulation sets the standard and criteria for a qualified electronic signature. A qualified electronic signature is defined as “*an advanced electronic signature that is created by a qualified electronic signature creation device, and which is based on a qualified certificate for electronic signatures*”, and is the legal equivalent of a handwritten signature (26). One of these three different types of signatures could be used to document IC via an electronic device, considering the requirements set out by this Regulation.

### The Acceptance of Electronic Informed Consent

Aside from the requirements of the European legislation that need to be complied with, requirements at the national level need to be considered when implementing eIC in clinical research. Therefore, we shed light on the legal acceptance of eIC in several EU Member States, Switzerland, and the United Kingdom. Generally, three groups of countries were identified. The first and second groups refer to countries that accept the use of eIC (including electronic signatures), with or without explicitly regulating it. The third group includes countries that do not accept the use of eIC (including electronic signatures). We provided examples for each of these groups. In addition, we compared the legal acceptance of eIC in the EU and the US (Table 2).

**TABLE 2:** The acceptance of electronic informed consent in the European Union and United States.

EU Member States			United States

eIC is allowed		eIC is not allowed	eIC is allowed
Group 1:	Group 2:	Group 3:	Regulated and clarified by a regulatory body
Explicitly regulated	Not regulated	Explicitly regulated	
(e.g., Belgium)	(e.g., Finland)	(e.g., Switzerland)	

*eIC, electronic informed consent; EU, European Union.*

#### Across European Union Member States, Switzerland, and the United Kingdom

##### Group 1: Countries That Regulate and Accept the Use of Electronic Informed Consent (Including Electronic Signatures) in Clinical Research

###### Austria

According to the Austrian Medicines Act Article 39(2), participants must provide IC in written form to participate in a clinical trial (27). Nevertheless, Article 4(1) of the Federal Act on Electronic Signatures and Trust Services for Electronic Transactions (Signature and Trust Services Act) describes that “*a qualified electronic signature satisfies the legal requirement of written form as defined in §886 of the General Civil Code*”, meaning that a qualified electronic signature is allowed to document the participants’ eIC (28). Moreover, the Austrian Federal Office for Safety in HealthCare specifies that electronic information, such as audio and video, can be used to inform participants on the condition that it is approved by an ethics committee (29).

###### Belgium

In Belgium, the conduct of clinical trials is regulated by the Law of May 7, 2004 on experiments on human beings. Pursuant to Article 6(1) of this Law, the participants’ consent must be given in writing (30). In addition, the use of electronic signatures is governed by the Law of July 21, 2016 on eIDAS and electronic archiving, which further implements the EU eIDAS Regulation (31). The Clinical Trial College, an independent body within the Federal Public Service Health, Food Chain Safety and Environment issued a guidance on the use of eIC in interventional clinical trials in Belgium. This guidance summarizes the following requirements: informing participants, signing consent forms, access to eIC after signing, the dossier to be submitted to the ethics committee, and the application of the GDPR. For example, this guidance specifies that the electronic method of signing the IC must be adapted to the clinical trial, the context of the IC process as well as the participants’ needs. If it concerns a phase 1 trial that requires the participants to show their official identity card at each visit, electronic signatures can be used that “*involve participant’s handwritten signature using a finger or a stylus or biometric e-signature under the condition that there is a table trail which makes it possible to demonstrate that the person making the electronic signature was indeed the participant (e.g., the check of the ID document of the person)*”. In addition, this guidance sets out that if the participants’ identity is not verified, an advanced or a qualified electronic signature must be used (32).

###### United Kingdom

The United Kingdom Health Research Authority (HRA) and Medicines and Healthcare products Regulatory Agency (MHRA) issued a joint statement, setting out the ethical and legal requirements when using eIC. In this statement, reference is made to the Medicines for Human Use (Clinical Trials) Regulations 2004 which lay down the requirements on informing and documenting consent in clinical trials. In addition, this statement clarified that electronic methods may be used for seeking, confirming, and documenting IC in research studies (33).

With regard to electronic signatures, the EU eIDAS Regulation is supplemented by the United Kingdom eIDAS Regulations (Statutory Instrument 2016/696) (34). Electronic signatures can be classified as “simple”, “advanced” or “qualified”. The type of electronic signature that should be used depends on the specific study. In the case of clinical trials of investigational medicinal products involving risks no higher than that of standard medical care, also referred to as type A trials, any simple electronic signature may be used (including typewritten or scanned eSignatures). In the case of type B and C trials involving a somewhat higher or a markedly higher risk compared to standard medical care, respectively, simple electronic signatures “*that involve the participant tracing their handwritten signature using a finger or a stylus or biometric eSignatures should normally be used as these allow for direct comparison with eSignatures and/or wet-ink signatures previously used by the participant for the purpose of audit or where the consent is contested*”. However, the HRA and MHRA advise against the use of typewritten or scanned images of handwritten signatures. When clinical trials are conducted remotely, it may not always be possible to verify the participants’ identity face-to-face. In these cases, the authorities prefer the use of advanced or qualified electronic signatures. In other types of research, any form of simple electronic signature should be sufficient to document consent. However, the HRA and MHRA emphasized that signatures traced with a finger or a stylus or biometric electronic signatures may be preferable for studies involving more than minimal risk, burden or intrusion (33).

###### Netherlands

Medical scientific research, involving participants who are subject to procedures or are required to follow rules of behavior, is subject to the Medical Research Involving Human Subjects Act (WMO) (35, 36). In 2020, a legislative procedure was initiated aiming to include the use of electronic signatures for obtaining the participants’ IC in the WMO (37). This amendment will enter into force at a time to be determined by Royal Decree. Article 6 of the WMO will be modified and will include that the participants’ IC “*can be obtained by electronic means, on the condition that such means are sufficiently reliable and confidential, appropriate to the research, and are set forth in the research protocol*”. In addition, Article 6 will lay down that the research participants shall be informed in the same manner, if possible and preferred by the participants, as in which consent can be given. In any case, information must be conveyed in writing and, if desired, in an interview with the research team (38).

##### Group 2: Countries That Accept the Use of Electronic Informed Consent (Including Electronic Signatures) in Clinical Research Without Explicitly Regulating It

###### Finland

Although eIC is not mentioned in the national legislation, the use of eIC in clinical research is possible. Cases are assessed individually by the Finnish Medicines Agency (FIMEA), which is the competent national authority for regulating pharmaceuticals (39). According to FIMEA, researchers must describe in their application how they would organize the eIC process. Based on that, applicants may have permission to use eIC.

The National Committee on Medical Research Ethics (TUKIJA) in Finland, an expert group on research ethics, advises regional ethics committees on ethical principles related to medical research (40). The TUKIJA has issued guidance and templates on IC for participation in clinical trials. In these documents, the Committee has clarified that eIC is also accepted: “*Written consent can also be provided electronically. If you intend to use an online system for obtaining consent, please provide a description of the method and your reasons for opting for this alternative in your application to the committee”* (41).

Furthermore, the Finnish National Health Information system (KANTA) allows Finnish citizens to access their electronic prescriptions, medical records and manage their consent online for several purposes (42, 43). These purposes might include secondary use of data for medical research. The Act on the Secondary Use of Health and Social Data (552/2019) allows the further use of health data for medical research through the Social and Health Data Permit Authority (FINDATA) (44, 45). The Authority’s jurisdiction on data permits and requests is based on section 44 of the Act on the Secondary Use of Health and Social Data (552/2019) (44). However, this Act applies to registry-based research, not to clinical trials. Register-based research “*utilizes health and social data for other purposes than for which the data was originally saved in the customer register or utilizes national registries*” (46). Although FINDATA and KANTA are not supporting clinical trials yet, the regulation and establishment of complex online health data management systems may help to foster the acceptance and trust in health cloud systems and eIC in the case of clinical trials.

##### Group 3: Countries That Do Not Accept the Use of Electronic Informed Consent (Including Electronic Signatures) in Clinical Research

###### Switzerland

The Swiss Ethics Committees on research involving humans issued a guideline on the use of eIC in clinical trials. According to this guideline, information can be conveyed by using electronic media, such as video or podcasts. The Swiss Ethics Committees recommend that “*the investigator, project leader, or the sponsor of the research project discuss plans for using an eIC with the Ethics Committee prior to finalizing the development of the eIC to ensure that the Ethics Committee agrees that the format may be used to convey the information the subjects*”. Nevertheless, a hand-written signature of the trial participants is necessary to document their consent. At the time of writing, the legal validity of electronic and/or digital signatures is under review. In addition, other requirements are set out in this guidance that must be taken into account when developing and using eIC. For example, it is required that “*a validated system is in place to ensure subject’s privacy, when electronic communication tools are used as part of the eIC interview process*” (47).

#### In the United States

In the US, there is no comprehensive, national data protection law. However, there are several sector-specific privacy and data security laws at federal, state, and local levels (48). At the federal level, the Health Insurance Portability and Accountability Act of 1996 (HIPAA) Privacy Rule aims to strike a balance between limiting the disclosure of personal health information and allowing researchers to access health data to support medical research. The disclosure of protected health information for research is allowed when: (i) the individual provides written consent or (ii) the Privacy Rule requires or permits it in other ways, such as approved by an institutional review board (49, 50). In the US, eIC is allowed and thoroughly regulated. The requirements for eIC are set forth by the US Food and Drug Administration (FDA) and the Department of Health and Human Services (HHS). More concretely, the relevant FDA and HHS regulations are outlined in the Code of Federal Regulations (CFR), under titles 21 and 45 (51, 52). Requirements related to IC, including the elements the participants need to be informed about, are presented in 21 CFR part 50 (53). Similarly, requirements related to documentation of IC are described in 45 CFR part 46. This part also addresses the elements that need to be included when broad consent is sought for the storage, maintenance, and secondary research use of identifiable information or samples (54). The use of electronic signatures is subject to 21 CFR part 11. This part sets out the criteria under which electronic signatures are considered equivalent to paper-based signatures. An electronic signature is defined as “*a computer data compilation of any symbol or series of symbols executed, adopted, or authorized by an individual to be the legally binding equivalent of the individual’s handwritten signature*”. The CFR clarifies the criteria of electronic signatures by detailing rules on the components, controls, integrity, and safety of electronic signatures, which are also applicable to clinical trials and biobanking (Table 3; 53).

**TABLE 3:** The regulation of electronic informed consent in the United States and the European Union.

	US	EU
Presentation of study-related information	HIPAA	CTR/GDPR
	21 CFR part 50	
	45 CFR part 45	
Electronic signatures	21 CFR part 11	eIDAS Regulation

*CFR, Code of Federal Regulations; CTR, Clinical Trials Regulation; eIC, electronic informed consent; eIDAS, electronic identification and trust services; EU, European Union; GDPR, General Data Protection Regulation; HIPAA, Health Insurance Portability and Accountability Act of 1996; US, United States.*

Moreover, in 2016, the FDA and HHS issued a guidance on eIC, intended for institutional review boards, investigators, and sponsors engaged in or responsible for oversight of human subject research under HHS. The guidance clarifies eIC as *“the use of electronic systems and processes that may employ multiple electronic media, including text, graphics, audio, video, podcasts, passive and interactive Web sites, biological recognition devices, and card readers, to convey information related to the study and to obtain and document informed consent”* (52). This clarification and further guidance on eIC are crucial for the unified application of rules on electronic signatures and IC. Overall, the requirements of electronic signatures and the IC process are regulated on the federal level in the US, resulting in a harmonized legal environment for acquiring eIC in the fields of healthcare and medical research.

## Discussion and Recommendations

Our manuscript provided an overview of the most important regulatory instruments relevant to eIC in clinical research. Although in the EU several regulations are regulating (parts of) eIC in clinical research, Member States still have room for introducing diverse requirements. The CTR, laying down the principles for IC for trial participation, does not set out requirements specifically related to eIC (17). The GDPR has strict rules on data protection, and allows the use of eIC, but does not require it (6). In addition, the eIDAS Regulation is not necessarily targeting the use of electronic signatures in clinical research (26). Some countries, accepting the use of electronic signatures, refer in their statements to the eIDAS Regulation while others, such as Switzerland, still require a wet-ink signature to document the participants’ consent. On the contrary, in the United States, the CFR lays down the requirements for eIC, and the FDA considers electronic signatures equivalent to handwritten, paper-based signatures. Overall, the regulations in the EU on data protection, clinical trials and electronic signatures result in a complex, partially harmonized legal environment, which poses a significant challenge for researchers implementing eIC.

Official position statements on the regulatory acceptance of eIC across EU Member States are highly variable. Some countries such as Austria and the United Kingdom published comprehensive guidance, allowing the use of eIC (including electronic signatures) in clinical research, whereas others such as Switzerland forbid it (29, 33, 47). Since drug development has been globalized to make treatments available to patients around the world, it may be crucial to harmonize the legal requirements across the EU Member States (55–57). Moreover, the EMA published a statement to urge the conduct of large multi-center, multi-arm clinical trials to investigate COVID-19 treatments. This statement stressed the importance of involving all EU Member States in these trials (58). When multi-country clinical trials are conducted, it needs to be ensured that the regulatory requirements in these countries are met. Due to the lack of harmonized eIC requirements, significant costs and additional delays may be added to the process of clinical research (59). Therefore, efforts are required to ensure that requirements have a high level of consistency to facilitate the conduct of multi-country clinical trials. Over the years, progress has been made in drafting guidance documents for eIC. In 2016, the FDA issued a guidance document in the US (52). In the EU, the EMA has taken the necessary steps to foster the adoption of eIC in clinical research by publishing a draft guideline on computerized systems and electronic data in clinical trials (14). These guidelines may contribute to increasing the regulatory convergence at a global level.

Requirements stemming from various regulations need to be considered during the implementation and use of eIC. When participants’ eIC is sought for research participation, the rules laid down by the CTR must be met. For instance, the eIC interface must inform participants about the aspects of the research study that are relevant to their decision on participation (17). An eIC system could make use of telemedicine technology to interactively guide participants through the information (8). In addition, the CTR specifies that obtaining participants’ written, dated and signed IC is a condition for a valid IC (17). If IC is documented via an electronic device, several electronic signature types could be used, as outlined by the eIDAS Regulation. However, a qualified electronic signature is the only type that has an equivalent value as a wet-ink signature (26). Next to IC for participation in research as an ethical requirement, it can also serve as one of the legal grounds for the processing of personal data. In this case, the eIC system must comply with the multiple obligations imposed by the GDPR. For example, similar to the CTR, the eIC interface must offer participants the possibility to withdraw their consent related to data processing (6).

## Conclusion

The application of eIC is increasingly becoming part of the clinical trial landscape, due to the COVID-19 outbreak and technological advancements. Therefore, our research outlined the regulatory framework relevant to eIC in clinical research, focusing on the EU. Although eIC has the potential to provide a safe, fast, and reliable tool to expedite research, the regulation of it is lagging behind, pulling back its potential. In the EU, despite the efforts to harmonize the rules on data protection and clinical trials, the legal acceptance of eIC significantly differs among the Member States. As our research highlighted, some Member States allow eIC (including electronic signatures), with or without explicitly regulating it, while other States simply do not allow the application of eIC, resulting in an unharmonized and confusing legal environment for researchers. In the United States, the acceptance and regulation of eIC on the federal level enables researchers to use the full potential of this technological application to enroll participants and have an interactive relationship with them during the research study. However, the sectorial approach and less strict rules on data protection rules are less efficient and result in an unharmonized protection in the US. Alignment of the regulatory requirements on eIC and data protection rules across countries may successfully advance the adoption of eIC, which would be crucial to enhance clinical research and successfully fight against present and future pandemics.

## Author Contributions

ED and JM designed and conducted this study and produced the first draft. PB and IH was subsequently revised the manuscript. All authors approved the final manuscript.

## Conflict of Interest

The authors declare that the research was conducted in the absence of any commercial or financial relationships that could be construed as a potential conflict of interest.

## Publisher’s Note

All claims expressed in this article are solely those of the authors and do not necessarily represent those of their affiliated organizations, or those of the publisher, the editors and the reviewers. Any product that may be evaluated in this article, or claim that may be made by its manufacturer, is not guaranteed or endorsed by the publisher.

## Footnotes

1 GDPR Article 6(1)(a).

2 GDPR Article 4(1).

3 GDPR Recital 32.

4 CTR Article 93.

5 CTR Article 28(1)(d).

6 GDPR Recital 161.

7 GDPR Article 6(1)(c).

8 GDPR Article 9(2)(i) allows the processing of personal data when this is “*necessary for reasons of public interest in the area of public health, such as [*…*] ensuring high standards of quality and safety of healthcare and of medicinal products or medical devices, on the basis of Union or member State law, which provides for suitable and specific measures to safeguard the rights and freedoms of the data subject, in particular professional secrecy.*”

9 CTR Article 58.

10 CTR Articles 77–79.

11 GDPR Article 6(1)(a) in conjunction with Article 9(2)(a).

12 GDPR Article 6(1)(e).

13 GDPR Article 6(1)(f) in conjunction with Article 9(2)(i) or (j).

14 CTR Article 28.

15 GDPR Article 9(2)(a).

16 GDPR Recital 43.

17 CTR Recital 31.

18 CTR Recital 76.

19 CTR Article 28(3).

20 GDPR Article 7(3).

21 GDPR Article 7(3).

22 eIDAS Regulation Recitals 13, 17, and 21.

23 eIDAS Regulation Article 26.
